# Changes of Body Weight and Body Composition in Obese Patients with Prader–Willi Syndrome at 3 and 6 Years of Follow-Up: A Retrospective Cohort Study

**DOI:** 10.3390/jcm9113596

**Published:** 2020-11-08

**Authors:** Giorgio Bedogni, Graziano Grugni, Sabrina Cicolini, Diana Caroli, Sofia Tamini, Alessandro Sartorio

**Affiliations:** 1 Clinical Epidemiology Unit, Liver Research Center, 34149 Basovizza, Italy; 2 Division of Auxology, Istituto Auxologico Italiano, IRCCS, 28824 Verbania, Italy; g.grugni@auxologico.it (G.G.); sartorio@auxologico.it (A.S.); 3 Experimental Laboratory for Auxo-Endocrinological Research, Istituto Auxologico Italiano, IRCCS, 28824 Verbania, Italy; s.cicolini@auxologico.it (S.C.); d.caroli@auxologico.it (D.C.); s.tamini@auxologico.it (S.T.)

**Keywords:** Prader–Willi syndrome, cohort study, weight loss, body composition, dual-energy X-ray absorptiometry, resting energy expenditure, indirect calorimetry, metabolic syndrome

## Abstract

Few short-term studies of weight loss have been performed in adult patients with Prader–Willi syndrome (PWS) undergoing metabolic rehabilitation. We performed a retrospective cohort study of 45 adult obese PWS patients undergoing a long-term multidisciplinary metabolic rehabilitation program based on diet and physical activity. Body composition was evaluated by dual-energy X-ray absorptiometry in 36 (80%) patients. The mean (95% CI) weight change was −3.6 (−7.6 to 0.4, *p* = 0.08) kg at 3 years and −4.6 (−8.5 to −0.8, *p* = 0.02) kg at 6 years, and that of BMI was −1.7 (−3.4 to 0.1, *p* = 0.06) kg/m^2^ at 3 years and −2.1 (−3.8 to −0.4, *p* = 0.02) kg/m^2^ at 6 years. A decrease of about 2% in fat mass per unit of body mass was observed, which is in line with the expectations for moderate weight loss. A possibly clinically relevant decrease in total and low-density lipoprotein cholesterol was also observed. These long-term results are important for patients with PWS, which is characterized by severe hyperphagia, behavioral disturbances, and cognitive impairment and is generally considered “resistant” to classical weight loss interventions.

## 1. Background

Prader–Willi syndrome (PWS) is a multisystemic disorder caused by lack of expression of genes on the paternally inherited chromosome 15q11.2-q13 region [1]. PWS, which has a birth incidence ranging from 1:10,000 to 1:30,000 and a population prevalence ranging from 1:10,000 to 1:134,000, affects both sexes equally and all ethnic groups [2,3,4]. PWS is characterized by hyperphagia and childhood-onset morbid obesity [5], which is likely to contribute substantially to the high mortality rate associated with the disorder [6]. Dysmorphic signs, multiple endocrine abnormalities, and cognitive and behavioral disturbances are other cardinal features of PWS [7].

Obesity associated with PWS is the most common syndromic obesity and is characterized by a peculiar body composition [8,9,10,11]. Subjects with PWS have, in fact, not only an expanded fat mass (FM) but also a lower fat-free mass (FFM) when compared to age- and sex-matched obese subjects without PWS. The resting energy expenditure (REE) of subjects with PWS is therefore lower, even if it is similar to that of subjects with simple obesity when it is standardized on FFM [12,13]. Importantly, for rehabilitation programs based on lifestyle changes, the total energy expenditure (TEE) of subjects with PWS is usually decreased because of reduced activity energy expenditure [14].

Lifestyle changes are central to the achievement of weight loss in obese persons with PWS [15], as they are in obese persons without PWS [16]. Lifestyle changes are however more difficult to achieve in PWS patients owing to their severe hyperphagia and behavioral disturbances [7]. Diet is usually offered to PWS patients as a part of a multidisciplinary intervention program involving physical activity and psychological support [17]. Nearly a decade ago, Grolla et al. pointed out that there were surprisingly few long-term follow-up studies of such intervention programs in adult patients with PWS [17].

This still holds true today, with just one study published in the last decade [18], while there is an increasing number of cohort studies and randomized controlled trials (RCT) using weight change as the primary outcome measure for pharmacological and bariatric interventions in patients with PWS, e.g., [19,20,21]. More studies of lifestyle changes are available for children than for adults with PWS [15,22], which, albeit understandable, does not allow to assess the effectiveness of lifestyle changes in adults with PWS. The rarity of PWS and the need for centers specialized in its treatment are the main reasons for the lack of such studies.

The main outcome of the present retrospective cohort study was to evaluate the changes in body weight at 3 and 6 years of follow-up in patients with PWS undergoing a multidisciplinary metabolic rehabilitation program at our center. During the study period, the patients were regularly followed every six months both as in-patients and out-patients.

## 2. Patients and Methods

### 2.1. Patients

We performed a retrospective cohort study of patients with PWS followed at the Division of Auxology of the Istituto Auxologico Italiano (Piancavallo, Verbania, Italy). The inclusion criteria were (1) genetically confirmed diagnosis of PWS; (2) age ≥ 17 years at baseline visit; (3) body mass index (BMI) ≥ 30 (kg/m^2^) at baseline visit; and (4) availability of anthropometric data (weight, height, and waist circumference), laboratory data (glucose, triglycerides, cholesterol, high-density lipoprotein (HDL) cholesterol, and low-density lipoprotein (LDL) cholesterol), clinical data (systolic and diastolic blood pressure), and indirect calorimetry data (REE) at baseline and at 3 (±0.5) and 6 (±0.5) years of follow-up. The data specified in point 4 above had to be collected before performing a planned 3-week in-hospital metabolic rehabilitation program. The study was approved by the ethical committee of the Istituto Auxologico Italiano (research project code: 01C921, acronym: FOLLOWUPPWS) and was conducted in accordance with the Declaration of Helsinki. Written informed consent to participate in the study was obtained from the patients aged ≥18 years or from the legal representatives of those aged <18 years.

### 2.2. Anthropometry

Weight and height were measured following international guidelines [23]. BMI was calculated as weight (kg)/height (m)^2^ and was classified according to the guidelines of the National Institutes of Health (NIH) [24]. Waist circumference was measured at the midpoint between the last rib and the iliac crest using an anthropometric tape [25].

### 2.3. Laboratory and Clinical Measurements

Glucose, triglycerides, total cholesterol, HDL cholesterol, and LDL cholesterol were measured by the same internal laboratory using standard methods. Blood pressure was measured using a sphygmomanometer following international guidelines. (The recommended method of measurement of blood pressure remained the same during the study period.) The metabolic syndrome (MS) was diagnosed using the criteria of the International Diabetes Federation (IDF) [26].

### 2.4. Indirect Calorimetry

REE was measured between 8:00 and 10:00 a.m. in thermoneutral conditions using a Sensor Medics Vmax 29 (Yorba Linda, CA, USA) metabolic cart equipped with a canopy, as described in detail elsewhere [27]. The subjects were in the fasting state for at least 8 h, had refrained from physical activity for at least 24 h, and had been waiting for at least 30 min in the sitting position before measurement. REE was measured in the supine position for at least 30 min, including an acclimation period of 10 min. The data relative to the acclimation period were discarded. REE was calculated from O_2_ consumption and CO_2_ production using the Weir equation [28].

### 2.5. Dual-Energy X-ray Absorptiometry

Body composition was measured using a dual-energy X-ray absorptiometry (DXA) (GE-Lunar Prodigy, GE Medical Systems, Milwaukee, WI, USA), as described in detail elsewhere [29]. Percent total FM (%) was calculated as (FM (kg)/body mass measured (BM) by DXA (kg)) × 100 and segmental FM (%) as (FM legs (kg)/FM (kg)) × 100, (FM arms (kg)/FM (kg)) × 100, and (FM trunk (kg)/FM (kg)) × 100 [10]. Fat-free mass (FFM) was obtained by subtracting FM from BM, and percent total FFM as (FFM (kg)/BM by DXA (kg)) × 100.

### 2.6. Multidisciplinary Metabolic Rehabilitation Program

The PWS patients performed a 3-week in-hospital multidisciplinary metabolic rehabilitation program at baseline and at 3 and 6 years of follow-up. All measurements analyzed here were collected before such in-hospital rehabilitation. During the study period, the patients were regularly followed every 6 months both as in-patients and out-patients. The metabolic rehabilitation program followed by obese patients with PWS at our center is similar to that followed by obese patients without PWS [30]. The in-patient program is based on two pillars: diet and exercise. A Mediterranean diet was prescribed in all cases, with an energy content obtained by subtracting at most 500 kcal from TEE, which is obtained by multiplying the measured REE by the level of physical activity [31]. The physical activity program consisted of 5 days of training per week and included: (1) 1 hour of moderately intense aerobic exercise with both arms and legs under the supervision of an instructor and, (2) from 20 to 30 min of cycloergometer exercise at 60 W or from 3 to 4 km of outdoor walking on flat terrain. At discharge from every visit, the patients and their caregivers received individualized counseling on nutrition and physical activity [32]. Lifestyle changes were promoted by the caring team for the duration of the study.

### 2.7. Statistical Analysis

Continuous variables are reported as median (50th percentile) and interquartile range (IQR, 25th and 75th percentiles). Discrete variables are reported as the number and proportion of subjects with the characteristic of interest. The mean (95% CI) changes of the outcomes of interest were estimated using a random-effects generalized linear regression model (RE-GLM). The RE-GLM employed a Gaussian family and an identity link, the continuous outcome (e.g., body weight, kg) as the response variable, time (discrete: 0 = baseline, 1 = 3 years, and 2 = 6 years) as the predictor, the patient as the random intercept, and time as the random slope [33]. Marginal means and 95% CI of the outcomes at all time points were estimated from the RE-GLM using the delta method [34]. Statistical analysis was performed using Stata 16.1 (Stata Corporation, College Station, TX, USA).

## 3. Results

Forty-five PWS patients, who were taken in by our center between June 2001 and February 2013, met the study entry criteria and were retrospectively studied. Table 1 reports the baseline measurements of these patients.

Twenty-eight (62%) PWS patients were women, and 31 (69%) had class III obesity. DXA measurements of body composition were available for 36 (80%) patients. Not surprisingly, the patients with DXA measurements were leaner than those without them. This was expected because the DXA scanner employed for the present study cannot accommodate subjects weighting more than 140 kg [29]. The prevalence of MS in the whole study population was 56% (25/45). No patient underwent treatment with anti-obesity drugs or bariatric surgery during the study.

Table 2 reports the changes in anthropometry, laboratory measurements, blood pressure, and REE for all patients (*n* = 45) and the changes in total and segmental body composition for patients with availability of DXA (*n* = 36, 80%).

The mean (95% CI) weight change was −3.6 (−7.6 to 0.4, *p* = 0.08) kg at 3 years and −4.6 (−8.5 to −0.8, *p* = 0.02) kg at 6 years. Even if the 95% CI is wide, its upper bound indicates weight loss in most cases. According to it, in fact, the worst-case scenario expected at 3 years for the population from which these patients are drawn is an increase of just 0.4 kg, and that expected at 6 years is a decrease of −0.8 kg. The corresponding mean (95% CI) BMI change is −1.7 (−3.4 to 0.1, *p* = 0.06) kg/m^2^ at 3 years and −2.1 (−3.8 to −0.4, *p* = 0.02) kg/m^2^ at 6 years. Appendix A gives the time-plots of weight at 6-month intervals for 2 patients with weight loss, 2 patients with stable weight, and 2 patients with weight gain at 6 years.

A possibly clinically relevant decrease in total cholesterol and LDL cholesterol was observed at 6 years, which is not explained by the use of cholesterol-lowering drugs. (One woman was taking a statin at baseline but not at 3 and 6 years, and another woman was under statin treatment at the 3- and 6-year follow-up visits.) Except for glucose, which showed an increasing trend possibly reflecting an underlying propension of PWS, the mean values of the remaining laboratory markers showed a favorable trend, i.e., increase of HDL cholesterol, decrease in triglycerides, and decrease in systolic and diastolic blood pressure. However, the precision of these estimates is low, as shown by their wide 95% CI, so that studies with larger sample sizes are needed to estimate whether they are clinically relevant or not.

REE did expectedly decrease with weight loss but remained constant per unit of weight. In the subsample of 36 (80%) PWS patients for whom DXA was available, there was a mean (95% CI) decrease in percent FM of −2.3% (−3.5% to −1.0%, *p* < 0.001) at 3 years and of −1.8% (−3.0% to −0.5%, *p* < 0.01) at 6 years.

Table 3 reports the absolute values of anthropometry, laboratory measurements, blood pressure, and REE for all patients (*n* = 45) and the absolute values of total and segmental body composition for patients with availability of DXA (*n* = 36, 80%). To understand the connection of Table 3 with Table 2, note that the intercept given in Table 2 corresponds to the mean baseline value given in Table 3 (RE-GLM).

## 4. Discussion

Nearly one decade ago, Grolla et al. pointed out that there were few long-term studies on the effectiveness of nutritional rehabilitation programs in obese adults with PWS [17]. They evaluated the effectiveness of a rehabilitation program at reducing body weight in 49 PWS patients with a median (IQR) age of 24 (17;28) years and a median (IQR) number of rehabilitation cycles of 2 (1;4), corresponding to a median (IQR) follow-up time of 0.5 (0.25;1) years (data calculated from the patient-level data given in Table 1 of [17]). On average, a rehabilitation cycle of this program was reported to produce a change of −2.1 kg/m^2^ in BMI [17]. A later study performed by Hauber and colleagues on 8 PWS patients with a median (IQR) age of 31 (27;36) years, reported a median (IQR) change of 1.65 (0.1;2.45) kg/m^2^ of BMI after 13 months of follow-up [18] (data calculated from the patient-level data given in Table 1 of [18]). No other published studies are available on the effectiveness of multidisciplinary nutritional rehabilitation programs in obese adults with PWS.

In the present study, we took advantage of the availability of three very detailed repeated in-hospital assessments to evaluate the 3- and 6-year longitudinal changes of body weight, body composition, REE, and cardiometabolic risk markers in 45 PWS patients. All measurements were taken before the three in-hospital metabolic rehabilitations. At 6 years, we found that the average weight loss was slightly less than 5% and that the average BMI loss (−2.1 kg/m^2^) was similar to that reported by Grolla et al. as an average of repeated measures for a median follow-up time of 0.5 years [17].

Our results are important for three reasons. The first reason is that the average weight loss observed in the present study may have clinical benefits, although this is currently proven only for people without PWS [35,36]. The second reason is that the average weight loss achieved by our PWS patients corresponds to the average weight loss achieved by individuals with primary obesity [37]. We believe that this is an important fact to tell the patients and their families, i.e., that they can reach, with the appropriate strategies, the same weight loss achieved by obese people without PWS. It should be added that most studies on the effect of lifestyle changes on non-syndromic obesity have a follow-up time shorter than 2 years [38]. The third reason is that persistent weight loss can be regarded as successful in PWS, which is characterized by severe hyperphagia, behavioral disturbances, and cognitive impairment and is generally labeled as “resistant” to weight loss interventions [14].

A decrease of about 2% in percent FM was observed at 6 years, which is in line with the expectations for moderate weight loss [39]. The change of percent FM was expectedly accompanied by the same increase in percent FFM. Concerning segmental body composition, we have previously reported that trunk fat tends to be lower in obese subjects with PWS than in those without PWS [10]. However, not surprisingly for the degree of weight loss observed in this study, the corresponding changes in segmental body fat were minor and of doubtful clinical relevance [39].

The prevalence of MS (56%) in the present study was higher than that reported by an Italian multi-center study (41%, 36/87) for obese patients with PWS [40]. This finding suggests that, despite recent progresses in the management of PWS [14], its metabolic complications do still need attention. Because of the relatively low number of subjects, as is to be expected for a rare disease such as PWS [40], and because of the intrinsic limitations of dichotomization, we studied the changes of the continuous outcomes defining MS instead of MS as a whole [41]. Among the cardiometabolic markers, a decrease in total and LDL cholesterol was observed, which may be clinically relevant and is not attributable to cholesterol-lowering drugs. Of course, much larger samples are needed to establish the clinical relevance of the change in cholesterol and other laboratory markers because of their expectedly imprecise estimates due to low sample size (Table 2). Just to allow comparison with other studies, we add that, using a RE-GLM as described under statistical analysis but with a Bernoulli family and a logit link [42], the prevalence of MS was estimated to be 56% (95% CI 41% to 71%) at baseline, 36% (22% to 51%) at 3 years, and 47% (31% to 62%) at 6 years, with an expectedly wide 95% CI owing to the low number of patients.

The present study has several strengths. First, it is the longest follow-up study (6 years) performed so far in PWS patients. Although 45 patients might not seem to be many, this number must be considered in light of the rarity of PWS and of the difficulty of obtaining outcome data spanning more than 2 years even in obese patients without PWS [38]. Second, all measurements, especially DXA and indirect calorimetry, were performed at a single center. Third, the metabolic rehabilitation program was performed by physicians and dietitians highly experienced with it [30].

The present study has nonetheless several limitations. The first limitation is that, being an observational study, it cannot prove any cause–effect relationship. Thus, although we observed a clinically relevant weight loss at 6 years, we cannot prove that it was produced by our rehabilitation program. RCTs using weight change as the main outcome are currently being employed to evaluate the effect of drugs and bariatric surgery on PWS-associated obesity, e.g., [19,20,43]. Our data suggest that some space should be left also for RCTs of lifestyle changes because they remain the central strategy in obtaining weight loss in obese persons with and without PWS [14,15,16]. The lack of a control group not performing the multidisciplinary intervention impedes, of course, comparison of the effects of our program with those of the natural course of disease. Ethical considerations advise against making such comparison because a proactive approach to lose weight is considered central to reducing mortality in PWS [44]. We can nonetheless make a comparison with a historical control group, an approach which has many methodological limitations but is the only viable option here. In an historical control group of 13 PWS patients (6 men and 7 women) with a mean (standard deviation, SD) age of 31 (11) years who did not take part to our metabolic rehabilitation program and had an irregular follow-up, mean (SD) BMI increased from 48.5 (8.0) to 54.3 (9.0) kg/m^2^ at 4 years, which is certainly clinically relevant [6]. The second limitation is that our findings were obtained in a tertiary care center with further specialization on PWS and may not be generalizable to other contexts. However, it is nowadays common to offer metabolic rehabilitation programs to persons with PWS inside specialized centers, and it is difficult to imagine a follow-up study such as the present one carried out outside such centers [17,18]. The third limitation is that body composition measurements were available only for 36 (80%) patients due to technical limitations of the DXA scanner employed for the present study [29]. Furthermore, because of the moderate weight loss, the changes of total and segmental body composition, albeit of great interest because of the peculiar body composition associated with PWS [8,9,10], were minor and greater weight losses are needed to disentangle the composition of weight loss in PWS.

## 5. Conclusions

In conclusion, patients with PWS undergoing a long-term multidisciplinary metabolic rehabilitation program show clinically relevant weight loss at 6 years of follow-up, which is accompanied by a loss of percent FM and by a decrease in total and LDL cholesterol. This long-term result is especially important for patients with PWS, which is characterized by severe hyperphagia, behavioral disturbances, and cognitive impairment and is generally considered “resistant” to classical weight loss interventions.

## Figures and Tables

**Table 1:** Measurements of the Prader–Willi syndrome (PWS) patients at baseline visit.

	DXA Available	DXA Not Available	All
*N*	36 (80%)	9 (20%)	45 (100%)
Karyotype			
DEL15	26 (72%)	7 (78%)	33 (73%)
UPD	10 (28%)	2 (22%)	12 (27%)
Sex			
Female	23 (64%)	5 (56%)	28 (62%)
Male	13 (36%)	4 (44%)	17 (38%)
Age (years)	25 (22; 30)	29 (25; 31)	26 (22; 30)
Weight (kg)	98.8 (83.8; 112.8)	132.5 (121.3; 144.3)	102.8 (85.0; 119.4)
Height (m)	1.51 (1.45; 1.57)	1.54 (1.52; 1.57)	1.52 (1.47; 1.57)
BMI (kg/m^2^)	43.6 (35.1; 47.6)	55.6 (49.5; 62.6)	44.6 (37.5; 52.0)
BMI class (NIH)			
Obesity class 1	9 (25%)	0 (0%)	9 (20%)
Obesity class 2	4 (11%)	1 (11%)	5 (11%)
Obesity class 3	23 (64%)	8 (89%)	31 (69%)
Waist circumference (cm)	119.5 (107.5; 125.5)	136.0 (113.0; 144.0)	121.0 (111.0; 131.0)
High waist circumference (IDF)			
No	1 (3%)	0 (0%)	1 (2%)
Yes	35 (97%)	9 (100%)	44 (98%)
Glucose (mg/dL)	84 (76; 96)	80 (79; 92)	83 (79; 96)
High glucose (IDF)			
No	30 (83%)	8 (89%)	38 (84%)
Yes	6 (17%)	1 (11%)	7 (16%)
Type 2 diabetes mellitus (IDF)			
No	29 (81%)	7 (78%)	36 (80%)
Yes	7 (19%)	2 (22%)	9 (20%)
Cholesterol (mg/dL)	195 (169; 210)	169 (141; 203)	194 (159; 207)
Treatment with cholesterol-lowering drugs (IDF)			
No	35 (97%)	9 (100%)	44 (98%)
Yes	1 (3%)	0 (0%)	1 (2%)
HDL cholesterol (mg/dL)	53 (40; 64)	40 (36; 44)	47 (38; 61)
Low HDL cholesterol (IDF)			
No	22 (61%)	1 (11%)	23 (51%)
Yes	14 (39%)	8 (89%)	22 (49%)
LDL cholesterol (mg/dL)	129 (106; 141)	111 (90; 126)	126 (105; 137)
Triglycerides (mg/dL)	90 (76; 110)	98 (58; 117)	91 (76; 111)
High triglycerides (IDF)			
No	32 (89%)	7 (78%)	39 (87%)
Yes	4 (11%)	2 (22%)	6 (13%)
Systolic blood pressure (mm Hg)	130 (120; 130)	120 (120; 130)	130 (120; 130)
Diastolic blood pressure (mm Hg)	80 (80; 80)	80 (80; 80)	80 (80; 80)
High blood pressure (IDF)			
No	16 (44%)	4 (44%)	20 (44%)
Yes	20 (56%)	5 (56%)	25 (56%)
Treatment with antihypertensive drugs (IDF)			
No	33 (92%)	3 (33%)	36 (80%)
Yes	3 (8%)	6 (67%)	9 (20%)
Metabolic syndrome score (IDF)			
0	1 (3%)	0 (0%)	1 (2%)
1	8 (22%)	0 (0%)	8 (18%)
2	9 (25%)	2 (22%)	11 (24%)
3	13 (36%)	4 (44%)	17 (38%)
4	5 (14%)	3 (33%)	8 (18%)
Metabolic syndrome (IDF)			
No	18 (50%)	2 (22%)	20 (44%)
Yes	18 (50%)	7 (78%)	25 (56%)
Cigarette smoking			
No	35 (97%)	8 (89%)	43 (96%)
Yes	1 (3%)	1 (11%)	2 (4%)
Treatment with growth hormone			
No	7 (78%)	26 (72%)	33 (73%)
Yes	2 (22%)	10 (28%)	12 (27%)
REE (kcal/day)	1624 (1409; 1893)	1856 (1792; 2000)	1754 (1435; 1907)
REE (kcal/day/kg body weight)	17 (16; 18)	15 (14; 17)	16 (15; 18)
FFM (kg)	53.2 (44.6; 62.4)	NA	NA
FFM (kg/kg BM, %)	50.4 (47.8; 56.1)	NA	NA
FM (kg)	49.8 (43.7; 59.1)	NA	NA
FM (kg/kg BM, %)	49.6 (43.9; 52.2)	NA	NA
FM arms (kg)	6.5 (4.6; 10.3)	NA	NA
FM arms (kg/kg FM, %)	13.5 (11.2; 18.6)	NA	NA
FM legs (kg)	18.3 (15.3; 21.3)	NA	NA
FM legs (kg/kg FM, %)	37.4 (33.6; 40.0)	NA	NA
FM trunk (kg)	24.5 (20.8; 27.2)	NA	NA
FM trunk (kg/kg FM, %)	47.0 (44.1; 52.6)	NA	NA

Continuous variables are reported as median (50th percentile) and interquartile range (IQR, 25th and 75th percentiles). Discrete variables are reported as the number and proportion of subjects with the characteristic of interest. Abbreviations: BMI = body mass index; BM = body mass; NIH = National Institutes of Health; IDF = International Diabetes Federation; REE = resting energy expenditure; NA = not available; FFM = fat-free mass; FM = fat mass.

**Table 2:** Changes of anthropometry, laboratory measurements, blood pressure, resting energy expenditure, and total and segmental body composition at baseline and at 3 and 6 years of follow-up.

	3rd Year vs. Baseline	6th Year vs. Baseline	Intercept ^†^
Weight (kg)	−3.6 (−7.6 to 0.4)	−4.6 * (−8.5 to −0.8)	104.4 *** (98.0 to 110.9)
BMI (kg/m^2^)	−1.7 (−3.4 to 0.1)	−2.1 * (−3.8 to −0.4)	45.4 *** (42.7 to 48.0)
Waist circumference (cm)	−2.4 (−6.9 to 2.1)	0.6 (−3.7 to 4.9)	121.6 *** (116.8 to 126.5)
Glucose (mg/dL)	3.5 (−3.8 to 10.7)	4.5 (−3.6 to 12.6)	90.7 *** (84.1 to 97.3)
Cholesterol (mg/dL)	−3.9 (−13.1 to 5.4)	−11.7 * (−20.6 to −2.7)	189.2 *** (179.0 to 199.4)
HDL cholesterol (mg/dL)	1.8 (−1.1 to 4.8)	1.3 (−1.6 to 4.3)	50.1 *** (46.1 to 54.1)
LDL cholesterol (mg/dL)	−2.8 (−10.6 to 5.1)	−8.1 * (−16.1 to −0.1)	123.9 *** (115.2 to 132.6)
Triglycerides (mg/dL)	−0.6 (−13.2 to 12.0)	3.9 (−8.3 to 16.1)	100.4 *** (87.8 to 113.0)
Systolic BP (mm Hg)	−3.0 (−7.7 to 1.7)	−1.2 (−5.7 to 3.4)	127.0 *** (123.6 to 130.4)
Diastolic BP (mm Hg)	−0.6 (−3.6 to 2.5)	−1.0 (−4.0 to 1.9)	80.4 *** (78.3 to 82.6)
REE (kcal/day)	−67.7 (−146.5 to 11.0)	−105.3 ** (−181.4 to −29.3)	1698.0 *** (1604.5 to 1791.5)
REE (kcal/kg weight/day)	−0.1 (−0.9 to 0.7)	−0.4 (−1.2 to 0.4)	16.6 *** (15.8 to 17.3)
FFM (kg) ^††^	1.1 (−0.7 to 3.0)	1.5 (−0.3 to 3.4)	54.2 (51.0 to 57.4)
FFM (kg/kg BM, %) ^††^	2.3 *** (1.0 to 3.5)	1.8 ** (0.5 to 3.0)	51.6 (50.2 to 53.0)
FM (kg) ^††^	−3.5 ** (−5.9 to −1.1)	−2.3 (−4.7 to 0.2)	51.1 *** (47.9 to 54.4)
FM (kg/kg BM, %) ^††^	−2.3 *** (−3.5 to −1.0)	−1.8 ** (−3.0 to −0.5)	48.4 *** (47.0 to 49.8)
FM arms (kg) ^††^	−1.0 (−2.4 to 0.4)	−1.5 * (−2.8 to −0.1)	7.9 *** (6.8 to 9.1)
FM arms (kg/kg FM, %) ^††^	−0.7 (−3.5 to 2.1)	−2.1 (−4.8 to 0.7)	15.2 *** (13.2 to 17.1)
FM legs (kg) ^††^	−1.5 * (−2.9 to −0.1)	0.0 (−1.4 to 1.4)	18.9 *** (17.4 to 20.5)
FM legs (kg/kg FM, %) ^††^	−0.4 (−2.4 to 1.5)	1.9 (−0.2 to 4.0)	37.0 *** (35.5 to 38.5)
FM trunk (kg) ^††^	−1.0 (−3.2 to 1.1)	−1.0 (−3.2 to 1.1)	24.1 *** (22.3 to 25.9)
FM trunk (kg/kg FM, %) ^††^	1.1 (−2.0 to 4.2)	0.3 (−2.9 to 3.5)	47.8 *** (45.7 to 50.0]

^†^ The intercept is the baseline mean estimated by random-effects linear regression (see Table 3). ^††^ Available for 36 (80%) of 45 patients (see Table 1). * *p* < 0.05, ** *p* < 0.01, *** *p* < 0.001. Values are means and 95% confidence intervals estimated by random-effects linear regression. Abbreviations: BMI = body mass index; BP = blood pressure; BM = body mass; REE = resting energy expenditure; FFM = fat-free mass; FM = fat mass.

**Table 3:** Values of anthropometry, laboratory measurements, blood pressure, resting energy expenditure, and total and segmental body composition at baseline and at 3 and 6 years of follow-up.

	Baseline	3rd Year	6th Year
Weight (kg)	104.4 (98.0 to 110.9)	100.9 (94.2 to 107.5)	99.8 (93.3 to 106.3)
BMI (kg/m^2^)	45.4 (42.7 to 48.0)	43.7 (41.0 to 46.4)	43.3 (40.6 to 46.0)
Waist circumference (cm)	121.6 (116.8 to 126.5)	119.3 (114.2 to 124.3)	122.3 (117.4 to 127.1)
Glucose (mg/dL)	90.7 (84.1 to 97.3)	94.1 (86.1 to 102.2)	95.2 (85.8 to 104.7)
Cholesterol (mg/dL)	189.2 (179.0 to 199.4)	185.3 (174.7 to 195.9)	177.5 (167.2 to 187.8)
HDL cholesterol (mg/dL)	50.1 (46.1 to 54.1)	51.9 (47.8 to 56.1)	51.4 (47.2 to 55.6)
LDL cholesterol (mg/dL)	123.9 (115.2 to 132.6)	121.1 (111.8 to 130.4)	115.8 (106.2 to 125.4)
Triglycerides (mg/dL)	100.4 (87.8 to 113.0)	99.8 (86.6 to 113.0)	104.3 (91.5 to 117.0)
Systolic BP (mm Hg)	127.0 (123.6 to 130.4)	124.0 (120.3 to 127.7)	125.8 (122.3 to 129.4)
Diastolic BP (mm Hg)	80.4 (78.3 to 82.6)	79.9 (77.6 to 82.1)	79.4 (77.3 to 81.6)
REE (kcal/day)	1698.0 (1604.5 to 1791.5)	1630.3 (1533.6 to 1726.9)	1592.7 (1498.2 to 1687.1)
REE (kcal/kg weight/day)	16.6 (15.8 to 17.3)	16.5 (15.7 to 17.3)	16.2 (15.4 to 16.9)
FFM (kg) ^†^	54.2 (51.0 to 57.4)	55.3 (52.1 to 58.6)	55.8 (52.5 to 59.0)
FFM (kg/kg BM, %) ^†^	51.6 (50.2 to 53.0)	53.9 (52.4 to 55.4)	53.4 (52.0 to 54.9)
FM (kg) ^†^	51.1 (47.9 to 54.4)	47.6 (44.2 to 51.0)	48.9 (45.5 to 52.2)
FM (kg/kg BM, %) ^†^	48.4 (47.0 to 49.8)	46.1 (44.6 to 47.6)	46.6 (45.1 to 48.0)
FM arms (kg) ^†^	7.9 (6.8 to 9.1)	6.9 (5.7 to 8.1)	6.5 (5.3 to 7.7)
FM arms (kg/kg FM, %) ^†^	15.2 (13.2 to 17.1)	14.5 (12.4 to 16.6)	13.1 (11.1 to 15.1)
FM legs (kg) ^†^	18.9 (17.4 to 20.5)	17.5 (15.9 to 19.0)	19.0 (17.4 to 20.6)
FM legs (kg/kg FM, %) ^†^	37.0 (35.5 to 38.5)	36.6 (34.8 to 38.3)	38.9 (36.9 to 40.9)
FM trunk (kg) ^†^	24.1 (22.3 to 25.9)	23.1 (21.1 to 25.0)	23.1 (21.1 to 25.0)
FM trunk (kg/kg FM, %) ^†^	47.8 (45.7 to 50.0)	48.9 (46.5 to 51.4)	48.2 (45.6 to 50.7)

^†^ Available for 36 (80%) of 45 patients (see Table 1). Values are means and 95% confidence intervals estimated by random-effects linear regression. Abbreviations: BMI = body mass index; BP = blood pressure; BM = body mass; REE = resting energy expenditure; FFM = fat-free mass; FM = fat mass.

**Figure A1:**
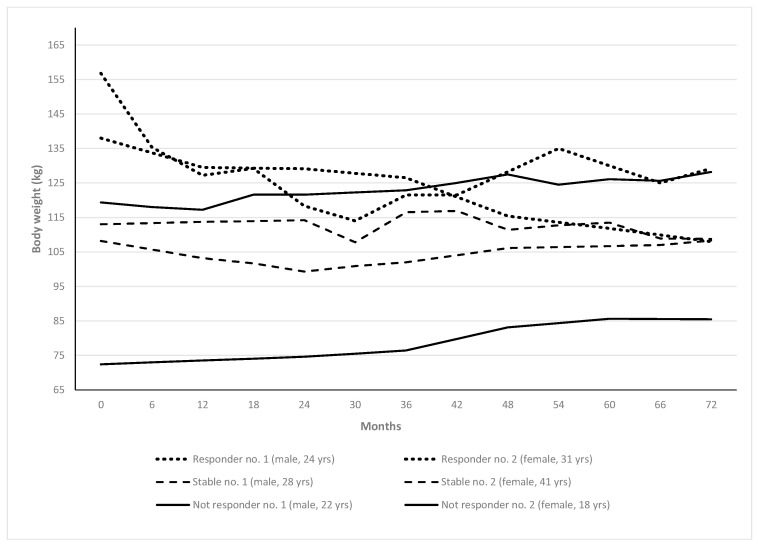
Time-plots of weight at 6-month intervals for 2 patients with weight loss (“responder”), 2 patients with stable weight (“stable”), and 2 patients with weight gain (“not responder”) at 6 years.

## References

[1] Butler M.G., Hartin S.N., Hossain W.A., Manzardo A.M., Kimonis V., Dykens E., Gold J.A., Kim S.J., Weisensel N., Tamura R. (2019). Molecular genetic classification in Prader-Willi syndrome: A multisite cohort study. J. Med. Genet..

[2] Cassidy S.B., Schwartz S., Miller J.L., Driscoll D.J. (2012). Prader-Willi syndrome. Genet. Med..

[3] Grugni G., Crino A., Bosio L., Corrias A., Cuttini M., De Toni T., Di Battista E., Franzese A., Gargantini L., Greggio N. (2008). The Italian National Survey for Prader-Willi syndrome: An epidemiologic study. Am. J. Med. Genet. A.

[4] Whittington J.E., Holland A.J., Webb T., Butler J., Clarke D., Boer H. (2001). Population prevalence and estimated birth incidence and mortality rate for people with Prader-Willi syndrome in one UK Health Region. J. Med. Genet..

[5] Miller J.L., Lynn C.H., Driscoll D.C., Goldstone A.P., Gold J.A., Kimonis V., Dykens E., Butler M.G., Shuster J.J., Driscoll D.J. (2011). Nutritional phases in Prader-Willi syndrome. Am. J. Med. Genet. A.

[6] Proffitt J., Osann K., McManus B., Kimonis V.E., Heinemann J., Butler M.G., Stevenson D.A., Gold J.A. (2019). Contributing factors of mortality in Prader-Willi syndrome. Am. J. Med. Genet. A.

[7] Angulo M.A., Butler M.G., Cataletto M.E. (2015). Prader-Willi syndrome: A review of clinical, genetic, and endocrine findings. J. Endocrinol. Invest..

[8] Forbes G.B. (1997). A distinctive obesity: Body composition provides the clue. Am. J. Clin. Nutr..

[9] Brambilla P., Bosio L., Manzoni P., Pietrobelli A., Beccaria L., Chiumello G. (1997). Peculiar body composition in patients with Prader-Labhart-Willi syndrome. Am. J. Clin. Nutr..

[10] Bedogni G., Grugni G., Tringali G., Marazzi N., Sartorio A. (2015). Does segmental body composition differ in women with Prader-Willi syndrome compared to women with essential obesity. J. Endocrinol. Invest..

[11] Bedogni G., Grugni G., Tringali G., Agosti F., Sartorio A. (2015). Assessment of fat-free mass from bioelectrical impedance analysis in obese women with Prader-Willi syndrome. Ann. Hum. Biol..

[12] Marzullo P., Mele C., Minocci A., Mai S., Scacchi M., Sartorio A., Aimaretti G., Grugni G. (2020). Fat-Free Mass Is Better Related to Serum Uric Acid Than Metabolic Homeostasis in Prader-Willi Syndrome. Nutrients.

[13] van Mil E.A., Westerterp K.R., Gerver W.J., Curfs L.M., Schrander-Stumpel C.T., Kester A.D., Saris W.H. (2000). Energy expenditure at rest and during sleep in children with Prader-Willi syndrome is explained by body composition. Am. J. Clin. Nutr..

[14] Crinò A., Fintini D., Bocchini S., Grugni G. (2018). Obesity management in Prader-Willi syndrome: Current perspectives. Diabetes Metab. Syndr. Obes..

[15] Miller J.L., Lynn C.H., Shuster J., Driscoll D.J. (2013). A reduced-energy intake, well-balanced diet improves weight control in children with Prader-Willi syndrome. J. Hum. Nutr. Diet..

[16] Raynor H.A., Champagne C.M. (2016). Position of the Academy of Nutrition and Dietetics: Interventions for the Treatment of Overweight and Obesity in Adults. J. Acad. Nutr. Diet..

[17] Grolla E., Andrighetto G., Parmigiani P., Hladnik U., Ferrari G., Bernardelle R., Lago M.D., Albarello A., Baschirotto G., Filippi G. (2011). Specific treatment of Prader-Willi syndrome through cyclical rehabilitation programmes. Disabil. Rehabil..

[18] Hauber M., Stratmann B., Hoedebeck-Stuntebeck N., Tschoepe D. (2013). Medical management for adults with Prader-Willi syndrome. Metab. Syndr. Relat. Disord..

[19] Scheimann A.O., Miller J., Glaze D.G. (2017). Laparoscopic sleeve gastrectomy in children and adolescents with Prader-Willi syndrome: A matched control study. Surg. Obes. Relat. Dis..

[20] Salehi P., Hsu I., Azen C.G., Mittelman S.D., Geffner M.E., Jeandron D. (2017). Effects of exenatide on weight and appetite in overweight adolescents and young adults with Prader-Willi syndrome. Pediatr. Obes..

[21] McCandless S.E., Yanovski J.A., Miller J., Fu C., Bird L.M., Salehi P., Chan C.L., Stafford D., Abuzzahab M.J., Viskochil D. (2017). Effects of MetAP2 inhibition on hyperphagia and body weight in Prader-Willi syndrome: A randomized, double-blind, placebo-controlled trial. Diabetes Obes. Metab..

[22] Lima V.P., Emerich D.R., Mesquita M.L., Paternez A.C., Carreiro L.R., Pina Neto J.M., Teixeira M.C. (2016). Nutritional intervention with hypocaloric diet for weight control in children and adolescents with Prader-Willi Syndrome. Eat Behav..

[23] Lohman T.G., Roche A.F., Martorell R. (1991). Anthropometric Standardization Reference Manual.

[24] National Institutes of Health (1998). Clinical Guidelines on the Identification, Evaluation, and Treatment of Overweight and Obesity in Adults. The Evidence Report. National Institutes of Health. Obes. Res..

[25] World Health Organization (2011). Waist Circumference and Waist-Hip Ratio: Report of a WHO Expert Consultation.

[26] Alberti K.G., Eckel R.H., Grundy S.M., Zimmet P.Z., Cleeman J.I., Donato K.A., Fruchart J.C., James W.P., Loria C.M., Smith S.C. (2009). Harmonizing the Metabolic Syndrome A Joint Interim Statement of the International Diabetes Federation Task Force on Epidemiology and Prevention National Heart, Lung, and Blood Institute American. Circulation.

[27] Bedogni G., Bertoli S., Leone A., De Amicis R., Lucchetti E., Agosti F., Marazzi N., Battezzati A., Sartorio A. (2019). External validation of equations to estimate resting energy expenditure in 14952 adults with overweight and obesity and 1948 adults with normal weight from Italy. Clin. Nutr..

[28] Weir J.B. (1949). New methods for calculating metabolic rate with special reference to protein metabolism. J. Physiol..

[29] Bedogni G., Grugni G., Tringali G., Tamini S., Marzullo P., Sartorio A. (2019). Assessment of fat-free mass from bioelectrical impedance analysis in men and women with Prader-Willi syndrome: Cross-sectional study. Int. J. Food Sci. Nutr..

[30] Rigamonti A.E., Cicolini S., Caroli D., De Col A., Scacchi M., Cella S.G., Sartorio A. (2020). Effects of a 3-Week In-Hospital Body Weight Reduction Program on Cardiovascular Risk Factors, Muscle Performance, and Fatigue: A Retrospective Study in a Population of Obese Adults with or without Metabolic Syndrome. Nutrients.

[31] Madden A.M., Mulrooney H.M., Shah S. (2016). Estimation of energy expenditure using prediction equations in overweight and obese adults: A systematic review. J. Hum. Nutr. Diet..

[32] Vismara L., Cimolin V., Grugni G., Galli M., Parisio C., Sibilia O., Capodaglio P. (2010). Effectiveness of a 6-month home-based training program in Prader-Willi patients. Res. Dev. Disabil..

[33] Rabe-Hesketh S. (2012). Multilevel and Longitudinal Modeling Using Stata. Volume I: Continuous Responses.

[34] Williams R. (2012). Using the Margins Command to Estimate and Interpret Adjusted Predictions and Marginal Effects. Stata J..

[35] Magkos F., Fraterrigo G., Yoshino J., Luecking C., Kirbach K., Kelly S.C., de Las Fuentes L., He S., Okunade A.L., Patterson B.W. (2016). Effects of Moderate and Subsequent Progressive Weight Loss on Metabolic Function and Adipose Tissue Biology in Humans with Obesity. Cell Metab..

[36] Ryan D.H., Yockey S.R. (2017). Weight Loss and Improvement in Comorbidity: Differences at 5%, 10%, 15%, and Over. Curr. Obes. Rep..

[37] Mancini J.G., Filion K.B., Atallah R., Eisenberg M.J. (2016). Systematic Review of the Mediterranean Diet for Long-Term Weight Loss. Am. J. Med..

[38] LeBlanc E.S., Patnode C.D., Webber E.M., Redmond N., Rushkin M., O’Connor E.A. (2018). Behavioral and Pharmacotherapy Weight Loss Interventions to Prevent Obesity-Related Morbidity and Mortality in Adults: Updated Evidence Report and Systematic Review for the US Preventive Services Task Force. JAMA.

[39] Müller M.J., Bosy-Westphal A. (2019). Effect of Over- and Underfeeding on Body Composition and Related Metabolic Functions in Humans. Curr. Diab. Rep..

[40] Grugni G., Crinò A., Bedogni G., Cappa M., Sartorio A., Corrias A., Di Candia S., Gargantini L., Iughetti L., Pagano C. (2013). Metabolic syndrome in adult patients with Prader-Willi syndrome. Nutr. Metab. Cardiovasc. Dis..

[41] Foschi F.G., Bedogni G., Domenicali M., Giacomoni P., Dall’Aglio A.C., Dazzani F., Lanzi A., Conti F., Savini S., Saini G. (2018). Prevalence of and risk factors for fatty liver in the general population of Northern Italy: The Bagnacavallo Study. BMC Gastroenterol..

[42] Rabe-Hesketh S. (2012). Multilevel and Longitudinal Modeling Using Stata. Volume II: Categorical Responses, Counts, and Survival.

[43] Kimonis V., Surampalli A., Wencel M., Gold J.A., Cowen N.M. (2019). A randomized pilot efficacy and safety trial of diazoxide choline controlled-release in patients with Prader-Willi syndrome. PLoS ONE.

[44] Manzardo A.M., Loker J., Heinemann J., Loker C., Butler M.G. (2018). Survival trends from the Prader-Willi Syndrome Association (USA) 40-year mortality survey. Genet. Med..

